# Quantifying the real life risk profile of inhaled corticosteroids in COPD by record linkage analysis

**DOI:** 10.1186/s12931-014-0141-y

**Published:** 2014-11-19

**Authors:** Rob W Flynn, Thomas M MacDonald, Adrian Hapca, Isla S MacKenzie, Stuart Schembri

**Affiliations:** Medicines Monitoring Unit, Ninewells Hospital and University of Dundee, Dundee, DD1 9SY UK; Tayside Respiratory Research Group, East Block, Ninewells Hospital, Dundee, DD1 9SY UK

**Keywords:** COPD, Inhaled steroids, Diabetes, Pneumonia, Cataract

## Abstract

**Background:**

Inhaled corticosteroids (ICS), especially when prescribed in combination with long-acting β_2_ agonists have been shown to improve COPD outcomes. Although there is consistent evidence linking ICS with adverse effects such as pneumonia, the complete risk profile is unclear with conflicting evidence on any association between ICS and the incidence or worsening of existing diabetes, cataracts and fractures. We investigated this using record linkage in a Dundee COPD population.

**Methods:**

A record linkage study linking COPD and diabetes datasets with prescription, hospitalisation and mortality data via a unique Community Health Index (CHI) number. A Cox regression model was used to determine the association between ICS use and new diabetes or worsening of existing diabetes and hospitalisations for pneumonia, fractures or cataracts after adjusting for potential confounders. A time dependent analysis of exposure comparing time on versus off ICS was used to take into account patients changing their exposure status during follow-up and to prevent immortal time bias.

**Results:**

4305 subjects (3243 exposed to ICS, total of 17,229 person-years of exposure and 1062 non exposed, with a follow-up of 4,508 patient-years) were eligible for the study. There were 239 cases of new diabetes (DM) and 265 cases of worsening DM, 550 admissions for pneumonia, 288 hospitalisations for fracture and 505 cataract related admissions. The hazard ratio for the association between cumulative ICS and outcomes were 0.70 (0.43-1.12), 0.57 (0.24-1.37), 1.38 (1.09-1.74), 1.08 (0.73-1.59) and 1.42 (1.07-1.88) after multivariate analysis respectively.

**Conclusion:**

The use of ICS in our cohort was not associated with new onset of diabetes, worsening of existing diabetes or fracture hospitalisation. There was however an association with increased cataracts and pneumonia hospitalisations.

## Background

Chronic obstructive pulmonary disease (COPD) causes significant morbidity and mortality and is the third leading cause of death worldwide [1]. The aim of COPD treatment is to mainly reduce symptoms [2] as no intervention other than smoking cessation and supplemental oxygen has consistently been shown to improve mortality [3,4].

Inhaled corticosteroids (ICS), especially when prescribed in combination with long-acting β_2_ agonists (LABA) improve quality of life (QoL), decrease exacerbations and hospitalisations, and have been associated with a trend towards a reduction in all-cause mortality [5]. However, unlike in asthma where the ICS dose is down titrated to the lowest possible that ensures symptom control, the ICS dose utilised in COPD can be quite high; a frequently used regime in the United Kingdom uses 500 μg fluticasone propionate twice daily [6]. A recent Cochrane review confirmed that ICS therapy is associated with increased pneumonic events [7]. However the effect of ICS on other complications such as osteoporotic fractures, onset and progression of diabetes, glaucoma and cataracts is less clear [8-11].

In order to determine which patients should be prescribed ICS, the adverse event profile of ICS must be properly defined. Knowledge of potential risks is especially important in situations where a drug is frequently used outwith the specific group of patients that it should be targeted at. This is especially the case with ICS, as for example in Scotland, the Scottish Medicines Consortium has consistently advised that ICS should not be used for patients with COPD and a FEV_1_ > 50% of predicted [12,13], however, they are nevertheless widely used in patients outside these strict spirometric parameters [14].

We aimed to investigate the association between use of ICS in COPD and development of incident diabetes or worsening of prevalent diabetes, and of other adverse events.

## Methods

This was a record-linkage cohort study using databases from Tayside Scotland. Data from the Tayside Medicines Monitoring Unit (MEMO) database is held within the Health Informatics Centre (HIC) [15]. This system collects data from the Tayside; a compact Scottish geographical area with a population of over 400,000 people. Health care for the region is co-ordinated by Tayside Health Board, which maintains a computerised record of all patients registered with a general practitioner (GP). In brief, the MEMO database contains several datasets including all dispensed community prescriptions, hospital discharge data, demographic data and biochemistry results. These data can be linked to disease-specific databases such as TARDIS (Tayside Allergy and Respiratory Disease Information System), DARTS (The Diabetes Audit and Research in Tayside Scotland; now called SCIDC) and other routine clinical data, all of which are linked by a Community Health Index (CHI) number that is unique to each patient.

### Data sources

*CHI master patient index* – this defined the study population from which subjects were identified, providing data on registered GPs and dates that patients got registered, together with patients' date of birth and date of death.

*MEMO prescription dataset* – this contains subject specific data on all prescriptions dispensed from community pharmacies in Tayside since 1993, including drug name, formulation, dosage, frequency and duration [15]. This provided data on the principal exposure of interest: ICS use. Other drug exposures used were other steroid use and anti-hypertensive medication.

*Scottish Morbidity Records 1 (SMR01)* – these data are routinely validated and collated by the Information and Services Division (ISD) of NHS Scotland and were available for Tayside from January 1, 1980 [15]. These contained diagnostic and procedural codes relating to all hospital inpatient episodes of care using the International Classification of Diseases ninth or tenth revisions (ICD-9, ICD-10) and Office of Population Censuses and Surveys Classification of Interventions and Procedures (OCSP4). These data were used to identify hospital admissions relating to pneumonia, fracture and cataract.

*TARDIS* –This has been described before [16]. GPs in Tayside refer patients with suspected COPD for screening spirometry. This is carried out in the GP practices by COPD nurses after structured training in order to obtain standardized results. COPD is diagnosed in patients with a FEV_1_ < 80% of the predicted value (greatest of pre- and post-bronchodilator values) and FEV_1_ /FVC <70%. Patients with COPD are then invited to participate in TARDIS, a structured management programme. Patients are seen annually and relevant measures recorded then.

*DARTS data* – this is a validated population-based clinical information system of patients (>8000) with diabetes in Tayside. Entries include attendances at hospital diabetes clinics, dispensed prescriptions for diabetes related medication and monitoring equipment, hospital discharge details, community-based mobile diabetic eye screening, glycosylated haemoglobin (HbA1c) and plasma glucose results from the regional biochemistry database. Validation against GP lists has confirmed DARTS to be robust [17].

### Study population

Subjects resident in Tayside and registered with a GP between January 2001 and December 2012. They were censored if they died or left Tayside during the study period.

### Study subjects

Subjects who registered with TARDIS database between January 2000 and December 2012 and who were 40 years old or over at diagnosis and who have at least two years of follow-up time formed the study cohort. The date of their first diagnosis of COPD (defined as having spirometry showing a FEV_1_/FVC <0.70) was used as the study entry date. Patients who had a cancer diagnosis prior to the diagnosis of COPD were excluded from the study. Patients who developed cancer during the follow up time were censored one year prior to the diagnosis of cancer. For the primary analysis patients with type-1 diabetes were excluded.

### Exposure

Each dispensed ICS prescription has details of date of prescription, daily dose, amount and duration. ICS exposure was converted into beclometasone equivalent doses [6].

## Outcomes

The primary outcome was either new cases of type 2 diabetes or worsening of pre-existing diabetes. These are defined below;

*Newly diagnosed diabetes –* new diagnosis of type 2 diabetes was recognised using the DARTS database, this has 95% sensitivity for identifying people with diabetes.

*Worsening of existing diabetic control –* defined as either worsening of HbA1c by >5 mmol/mol or the prescription of additional hypoglycaemic agents, following the index visit.

*Secondary endpoints –* hospitalisations coded as pneumonia, fractures and cataracts were obtained from SMR1. The relevant ICD10 admissions and OPCS4 procedural codes are listed in Table 1 [18,19].

**Table 1:** **Coding systems and specific codes used to identify study endpoints**

**Outcome**	**Coding system and codes used**
Pneumonia	ICD10: J12, J15, J16, J17, J18
Fractures	ICD10: S02, S12, S22, S32, S42, S52, S62, S72, S82, S92
Cataracts	OSCP4: C71, C72, C73, C74, C75
	ICD 10 code:H25

ICD10 - International Statistical Classification of Diseases 10th revision.

OSCP4 - Office of Population Censuses and Surveys Classification of Surgical Operations 4th revision.

### Covariates

Covariates considered in the analysis consisted of other factors that could be potential confounders. These were: (1) Demographics (age, sex, social deprivation score, smoking status and BMI); (2) Severity of COPD: (FEV_1_ and ratio of FEV1 /FVC at baseline, MRC dyspnoea score in the year before the first ICS use; (3) Disease history: cardiovascular risk defined as either primary or secondary prevention: primary prevention - hypertension (defined as on any anti-hypertension drug; treatment) and dyslipidaemia (defined as serum total cholesterol >5 mmol/L). Secondary prevention - myocardial infarction, heart failure, stroke, peripheral vascular disease (defined as hospitalisation from the SMR1 database). Renal disease was defined as having serum creatinine ≥220 μmol/l. For the secondary outcomes, a prior history of the outcome “event” at baseline was included in the model.

### Statistical analysis

Baseline characteristics were summarised as mean (SD)/median (IQR) for continuous variables and number of subjects (%) for categorical variables. These were compared using t-test for continuous variables and chi-square test for binary variables. A Cox regression model was used to explore the relationship between ICS exposure and the primary and secondary outcomes. Each endpoint was performed as a separate analysis allowing the calculation of relative risks associated with ICS use for each of the outcomes. A time dependent analysis of exposure – comparing time “on” versus “off” ICS exposure as judged by dispensed prescribing – was used to take into account the fact that patients changed their exposure status during follow-up and to prevent immortal time bias. The nature of the association between the exposure and outcome was incorporated into the time dependent model following that thought most biologically plausible: for cataract and osteoporosis this was cumulative steroid exposure; for pneumonia was current exposure; whilst for diabetes both cumulative and current exposure were modelled as there is no clear mechanism.

Both univariate and multivariate analyses were carried out. The multivariate analyses included all covariates thought to be potential confounders in an attempt to establish the true treatment effect. Variables included in the statistical models were potential predictors of the outcome events. Other covariates were evaluated once at the start of the follow up period. For the pneumonia outcome the population attributable risk was calculated by multiplying (hazard ratio-1)/hazard ratio by the probability of disease given exposure.

The exposed cohort was followed up from the date of first exposure to ICS. Non-ICS exposed patients were followed up from a randomly generated surrogate date of first “exposure” generated using a frequency-matched calendar year date of exposure. All statistical analyses were carried out using SAS (version 9.2).

There were very few missing data in the record linked data: (1.1% deprivation score, 4.4% BMI, 4.6% dyspnoea score). Where covariates did have missing values a complete case analysis performed. To establish if there were any relevant interactions in the multivariate analyses, two-way interactions terms were modelled incorporating ICS exposure and other key covariates of interest.

### Sensitivity analyses

A number of sensitivity analyses were carried out to test the robustness of the finding. First we used an inception cohort – consisting of only those patients naive to ICS at the start of follow-up.

Propensity score matching was used to explore possible confounding by indication that was not adequately controlled for by the Cox model. In view of data showing that the effect of ICS on pneumonia is no longer evident 6 months after drug cessation [20], analyses were also run using an extended screening period of 6-months to test the stability of the models generated and to see if there was any evidence of a carryover effect. The primary analysis considered any dose of ICS, versus none. As sensitivity analysis we also attempted to establish if there was a dose-response between exposure and outcome at zero, low, medium and high ICS doses. To standardise different doses of ICS we mapped potency as budesonide = beclametasone =50% fluticasone (i.e. 1mcg budesonide =1mcg beclometasone =0.5mcg fluticasone) [6]. This approach allowed the dose response of the different ICS products to be compared. Similarly this standardisation was also used to allow comparison of the different steroids, to see if it was possible to detect a difference between outcome and the type of steroid used (i.e. fluticasone vs “other” – beclometasone, budesonide etc).

To assess the impact of “other” (non-inhaled) steroid use, a sensitivity analysis was also conducted which stratified the cohort by extent of other steroid use. Finally we also repeated analyses using a fully time dependent analysis starting from point of diagnosis on TARDIS for all patients rather than the date of first exposure (or “surrogate date” of exposure as described).

## Results

**Figure 1:**
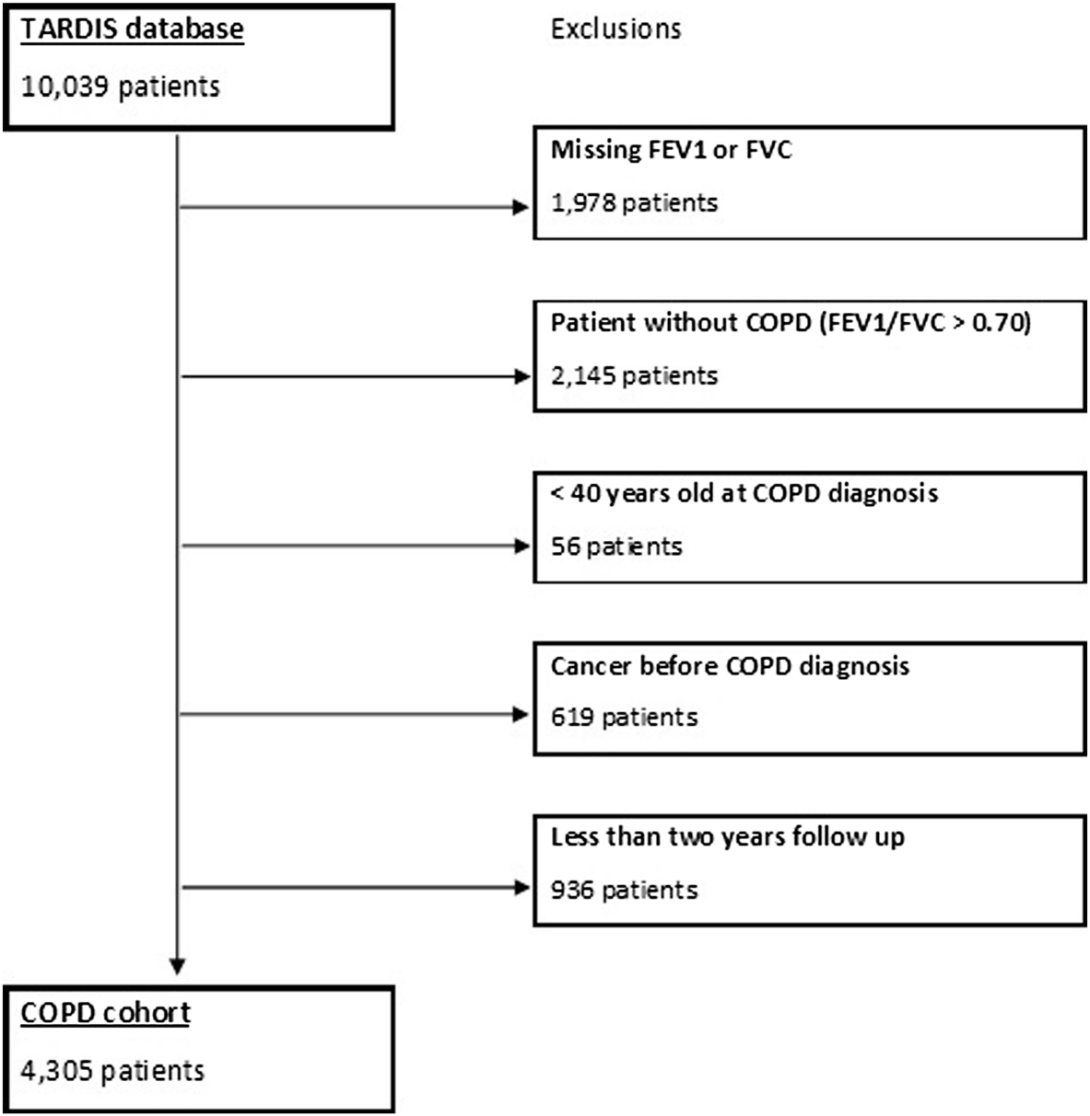
**CONSORT diagram showing derivation of study cohort.**

We identified 4,305 subjects as being eligible for the study cohort. Figure 1 details the number of patient available on the TARDIS database and details on excluded patients. 3,243 were exposed to ICS for a total of 17,229 person-years of exposure, and 1,062 were unexposed with a follow-up of 4,508 years. A comparison of the baseline characteristics of the exposed and unexposed cohorts is shown in Table 2. Fluticasone was responsible for 67.7% of the ICS prescription exposure and beclometasone for 20.5%. The remaining ICS prescriptions (11.8%) were for budesonide. There were 239 cases of newly diagnosed and 265 cases of worsening of existing diabetes. For the secondary outcomes there were 550 admissions for pneumonia, 288 hospitalisations for fracture and 505 cataract related admissions. The number of events by exposure group and exposure time is shown in Table 3. This shows the nature of the risk for the two components of the primary endpoint to be quite different, with the new onset diabetes being uncommon across a large number of patients, whilst the worsening of diabetes outcome was extremely common across a small number of patients. For this reason these two endpoints were treated separately for the remaining analyses.

**Table 2:** **Comparison of the baseline characteristics of the ICS exposed and unexposed cohorts**

**Continuous variables**	**ICS exposed**	**ICS un-exposed**	**p value** ^**#**^
	**(n = 3243)**	**(n = 1062)**	
Mean age (years)	65.5	67.2	< 0.0001
Mean BMI (kg/m2)	26.3	26.6	0.1772
Smoking (pack years)	34.7	39.0	< 0.0001
Social deprivation (Scottish index of multiple deprivation; 1 = most deprived, 5 = most affluent)	2.31	2.29	0.5972
Dyspnoea score (1 = least breathless, 5 = most breathless)	2.57	2.19	< 0.0001
Gender (female)	51.9%	49.5%	0.1693
Primary CV prevention (hypertension, dyslipidaemia)	73.4%	79.0%	0.0003
Secondary CV prevention (myocardial infarction, heart failure, stroke, PVD)	11.3%	15.6%	0.0002
Renal diseases history	2.9%	2.5%	0.4692
Diabetes history	8.0%	9.8%	0.0711
Pneumonia history	5.4%	3.8%	0.0375
Fracture history	5.7%	5.8%	0.8413
Cataract history	10.3%	10.9%	0.5840
Oral steroids prescription history	58.9%	30.0%	< 0.0001
Rectal steroids prescription history	11.8%	10.4%	0.1971
Topical steroids prescription history	61.1%	59.8%	0.4540
FEV_1_ baseline (Litres)	1.63 (0.65)	1.82 (0.63)	< 0.0001
Percent predicted FEV_1_^*^	74.9 (23.3)	85.0 (20.8)	< 0.0001
FEV_1_ /FVC ratio	55.1 (10.9)	59.8 (8.6)	< 0.0001

*“Percentage predicted” is baseline FEV_1_ expressed as percentage of predicted FEV_1._

^#^t-test for continuous variables and chi-square test for binary variables.

**Table 3:** **Number of events and year of follow-up for each of the study endpoints**

**Event**	**ICS exposed (n = 3243)**			**ICS unexposed (n = 1062)**		
	***n*** **events**	Person-years follow-up*	**Event rate (per 1000 person years)**	***n*** **event**	**Person-years follow-up**	**Event rate (per 1000 person years)**
New onset diabetes (*n* at risk =3,941)	186	16,634	11.2	53	4,285	12.4
Worsening of existing diabetes (*n* at risk =364)	194	595	326.1	71	223	318.4
Hospitalisation for pneumonia	476	17,795	26.8	74	4,833	15.3
Hospitalisation for fracture	234	18,097	12.9	54	4,822	11.2
Cataract	411	17,314	23.7	94	4,581	20.5

*”person years follow-up” is the number of years exposure / non-exposure during which subjects were at risk of suffering the event of interest. This varies depending on the number of subjects in the cohort and the number / timing of any events that occurred.

The results of the multivariate analyses are shown in Table 4. These show that there was no association between ICS use and either onset of new cases of diabetes or cases of worsening of existing diabetes. This was consistent across the whole range of sensitivity analyses that were run. For the secondary endpoints a consistent association was found between ICS and increased risk of both hospital admissions for pneumonia and cataract, but not fracture. The sensitivity analyses using models that incorporated various drug exposure screening periods found evidence of a carryover effect for the pneumonia outcome. This implies that the association between ICS use and hospitalisation for pneumonia continues to be present for some time after the ICS use has ceased (Tables 4 and 5). The population attributable risk associated with the use of ICS is an extra 7.4 pneumonia hospitalisation per 1,000 person years of exposure, with the results that of the 550 observed pneumonia hospitalisation in our study cohort 131 (23.8%) could be attributed to use of ICS. A statistically significant interaction between age and sex was found for the pneumonia outcome only; other interaction terms were not significant. Table 5 shows the association between fluticasone alone and other ICS with outcomes. This shows that there were no differences between steroid groups and incidence of diabetes or fractures however there are increased pneumonic and cataract hospitalisations in the patients treated with fluticasone. Analysing only the group of patients on ICS showed that fluticasone had a significantly increased risk of pneumonia hospitalisation (HRs no carryover 1.39 (1.06-1.82), 180 day carryover 1.43 (1.14-1.78)) when compared with individuals on other ICS.

**Table 4:** **Association between inhaled steroids and outcomes**

**Outcome**	**Univariate hazard ratio**	**Multivariate hazard ratio**	**Other variables contained in adjusted multivariate model**
	**(95% ** **CI)**	**(95% ** **CI)**	
**Primary endpoints**			
***New onset diabetes***			
Any exposure	0.88 (0.68-1.14)	0.94 (0.71-1.25)	Age*, sex, deprivation, history ICS, history oral steroids*, smoking, BMI*, primary CV history*, secondary CV history*, renal dysfunction, COPD severity
Cumulative exposure	0.71 (0.46-1.10)	0.70 (0.43-1.12)	Age*, sex, deprivation, history ICS, history oral steroid*, smoking, BMI*, primary CV history*, secondary CV history, renal dysfunction, COPD severity
***Worsening of existing diabetes***			
Current exposure	0.94 (0.73-1.21)	0.91 (0.73-1.21)	Age*, sex, deprivation, previous ICS, history of oral steroid use, smoking, BMI, primary CV history, secondary CV history, renal dysfunction, COPD severity*
Cumulative exposure	0.57 (0.25-1.30)	0.57 (0.24-1.37)	Age*, sex, deprivation, previous ICS, history of oral steroid, smoking, BMI, primary CV history, secondary CV history, renal dysfunction, COPD severity*
**Secondary endpoints**			
***Hospitalisation for pneumonia***			
Current exposure no carryover	1.27 (1.07-1.50)	1.13 (0.94-1.36)	Age*, sex, deprivation*, previous ICS, history of oral steroid, smoking*, BMI*, primary CV history*, secondary CV history*, renal dysfunction, COPD severity*, history of diabetes*, history of pneumonia admission*
Current exposure with 180-day carryover	1.58 (1.29-1.93)	1.38 (1.09-1.74)	
***Hospitalisation for fracture***			
Cumulative exposure	1.06 (0.75-1.51)	1.08 (0.75-1.51)	Age*, sex*, deprivation, previous ICS, history of oral steroid, smoking, BMI*, primary CV history, secondary CV history, renal dysfunction, COPD severity, history of diabetes, history of fracture admission*
***Cataract related outcome***			
Cumulative exposure	1.43 (1.11-1.83)	1.42 (1.07-1.88)	Age*, sex, deprivation, previous ICS, history of oral steroid, smoking*, BMI, primary CV history*, secondary CV history, renal dysfunction, COPD severity*, history of diabetes*, history of cataract related admission

*Shows variables that were found to be significant in the model.

*Abbreviations*: *CI* confidence interval, *ICS* inhaled corticosteroids, *BMI* body mass index, *CV* cardiovascular, *COPD* chronic obstructive pulmonary disease.

“COPD severity” is the actual FEV_1_ and a percentage of predicted FEV_1._

**Table 5:** **Comparison of effect of fluticasone and “other ICS” on outcomes**

**Outcome**	**Multivariate hazard ratio**		
	**(95% ** **CI)**		
	**No ICS**	**Other ICS**	**Fluticasone**
**Primary endpoints**			
***New case diabetes***	1.00	0.98 (0.69-1.39)	0.82 (0.58-1.15)
***Worsening of existing diabetes***	1.00	1.01 (0.72-1.41)	0.84 (0.60-1.17)
**Secondary endpoints**			
***Hospitalisation for pneumonia***			
No carryover	1.00	0.88 (0.68-1.14)	1.23 (1.01-1.51)
180-day carryover	1.00	1.05 (0.79-1.39)	1.50 (1.17-1.91)
***Hospitalisation for fracture***	1.00	1.08 (0.72-1.60)	1.10 (0.57-2.10)
***Cataract related outcome***	1.00	1.34 (0.99-1.82)	2.24 (1.40-3.60)

The extent of steroid use via all routes is shown in Table 6. This shows that there was widespread use of steroids administered by other routes. Indeed the majority of patient received greater exposure to oral than inhaled steroid. Use of rectal steroids was not common and the true extent to which systemic absorption of topical steroids is an issue is hard to assess. However the addition of oral steroid exposure to the models had little impact on the point estimates of the hazard ratios for the primary and secondary endpoints. The other sensitivity analyses yielded results that had no impact of the main finding of this study. The inception cohort analyses resulted in a sufficiently small cohort that results tended to be non-significant. The propensity score analyses showed nothing to suggest that they offered any additional adjustment for confounding. Similarly the other sensitivity analyses undertaken showed the study findings to be robust.

**Table 6:** **Mean and median cumulative doses of all steroid routes for all patients**

**Steroid exposure route**	**n**	**Mean**	**Median**	**Inter quartile range**
Cumulative inhaled dose	3,243	0.45 g	0.27 g	0.01-0.69 g
Cumulative oral dose	2,465	1.47 g	0.25 g	0-1.4 g
Cumulative rectal dose	257	0.01 g	0 g	0-0 g
Cumulative number of prescription for topical steroids	1,966	24	0	0-32

## Discussion

Our results show that the use of ICS in our cohort was not associated with new onset of diabetes or worsening of existing diabetes. Although systemic corticosteroids have a well recognised role in increasing diabetes risk [21], the impact of ICS is less well characterised and our study adds to the body of evidence in this field. Initial studies did not show an association between ICS use and diabetes [22,23], however a large population based study of 388,584 patients with respiratory disease (not just COPD) showed that ICS treatment was associated with a 34% increased risk of new onset diabetes (defined as initiation of an oral hypoglycaemic agent), with those on the highest ICS dose having the greatest risk [11]. More recently, a retrospective study of administrative claims data from the Australian Government Department of Veterans’ Affairs of more than 18,000 patients with diabetes, found there to be an increased risk of diabetes-related hospitalisations with the use of high-dose ICS [24]. Following this observational data, O’Byrne and colleagues examined double-blind, placebo-controlled, trials in patients ≥4 years of age with asthma or COPD involving budesonide or fluticasone to a lesser extent and did not find an association between inhaled corticosteroid treatment and increased risk of new onset diabetes mellitus or hyperglycaemia [25]. This is consistent with our findings and suggests that any effect of ICS on diabetes in COPD patients, if present at all, is unlikely to be clinically significant.

The relationship between ICS and pneumonia risk was first noted in TORCH with participants receiving ICS treatment having a two-fold higher rate of pneumonia when compared with those in the placebo arm [5]. These events were often diagnosed and managed in primary care, our results show that ICS use was also associated with hospitalisation for pneumonia. It has been suggested that ICS type may influence the risk of pneumonia, with a recent Cochrane review and studies such as PATHOS suggesting that fluticasone carries a higher risk than others such as budesonide; this difference is also seen in our study [7,26]. Fluticasone’s increased immunosuppressant potency (10-fold higher than that of budesonide with regard to *ex vivo* inhibition of human alveolar macrophage innate immune response to bacterial triggers) [27] could potentially explain these findings. As with other datasets we have shown that ICS use is associated with cataract surgery but not hospitalisation for fractures suggesting that there is no clinically significant association with osteoporosis [9,28]. Indeed a recent Cochrane review reported that ICS were not associated with an effect on fractures and bone mineral density [29] A previously unreported association was that fluticasone was more strongly associated with cataract surgery than other ICS, it is unclear why this may be the case however dose effect may be one explanation.

In order to justify prescribing ICS one must have data on the risk/benefit balance hence understanding the adverse effects of ICS is key. Although ICS have been shown to improve QoL, decrease exacerbations and hospitalisations and possibly improve mortality, evidence of benefit is limited to individuals with FEV_1_ < 70% predicted [30]. Studies that solely recruited patients with milder disease did not show similarly improved outcomes [31] however several studies have shown that over half of ICS use in daily practice falls outwith strict spirometric parameters included in COPD guidelines [32].

The strengths of this study are that data were collected in routine care with minimal exclusions: this increases the likelihood that the results will be applicable to other populations. Another strength is that all COPD diagnoses were validated using spirometric data, to our knowledge this is the first observational study on the effects of ICS and diabetes in COPD that has done this. Despite data being collected prospectively according to predefined criteria, follow-up data are observational and therefore prone to the weaknesses of this type of study. Though we attempted to limit bias by adjusting for other potential confounders, bias due to unrecorded factors may remain.

## Conclusions

ICS exposure in our cohort was not associated with new onset of diabetes, worsening of existing diabetes or fracture hospitalisation. There was however an association with increased cataracts and pneumonia hospitalisations. Knowing the adverse effect profile of ICS is especially important as they are often used in patients with no proven evidence of benefit.

## Abbreviations

ICS: Inhaled corticosteroids
COPD: Chronic obstructive pulmonary disease
CHI: Community health index
DM: Diabetes mellitus
LABA: Long-acting beta agonist
QoL: Quality of life
FEV1: Forced expiratory volume in one second
MEMO: Tayside medicines monitoring unit
HIC: Health informatics centre
GP: General practitioner
TARDIS: Tayside allergy and respiratory disease information system
DARTS: Diabetes audit and research in tayside
SMR01: Scottish morbidity records 1
ISD: Information and services division
ICD: the International classification of diseases
OCSP4: Office of population censuses and surveys classification of interventions and procedures (OCSP4)
HbA1c: Glycosylated haemoglobin
